# The Emory‐Sage‐SGC‐JAX TREAT‐AD Center: Target Enabling Packages for Alzheimer’s Disease Emerging Targets

**DOI:** 10.1002/alz.089250

**Published:** 2025-01-09

**Authors:** Karina Leal, Alison D. Axtman, Ranjita Betarbet, Paul E Brennan, Gregory W Carter, Haian Fu, Anna K Greenwood, Frank M. Longo, Aled M Edwards, Allan I. Levey, Emory‐Sage‐SGC‐Jax TREAT‐AD Center

**Affiliations:** ^1^ Sage Bionetworks, Seattle, WA USA; ^2^ University of North Carolina at Chapel Hill, Chapel Hill, NC USA; ^3^ Structural Genomics Consortium, Chapel Hill, NC USA; ^4^ Emory University School of Medicine, Atlanta, GA USA; ^5^ Alzheimer’s Research UK Oxford Drug Discovery Institute, University of Oxford, Oxford United Kingdom; ^6^ The Jackson Laboratory, Bar Harbor, ME USA; ^7^ Stanford University, Stanford, CA USA; ^8^ University of Toronto, Toronto, ON Canada; ^9^ Structural Genomics Consortium, Toronto, ON Canada

## Abstract

**Background:**

There is an urgent need for new therapeutic and diagnostic targets for Alzheimer’s disease (AD). Dementia afflicts roughly 55 million individuals worldwide, and the prevalence is increasing with longer lifespans and the absence of preventive therapies. Given the demonstrated heterogeneity of Alzheimer’s disease in biological and genetic components, it is critical to identify new therapeutic approaches. The Emory‐Sage‐SGC‐JAX TREAT‐AD Center is generating and openly distributing validated experimental tools necessary to test target predictions generated through sequence‐based characterization of human disease state. We believe that these tools and reagents, including chemical and biological probes that target the multifaceted dysregulation in the brains of AD patients, will advance the discovery of potential drug targets for AD.

**Method:**

Our Center has focused on developing a robust target prioritization and target enabling package (TEP) development pipeline, where we not only produced, characterized, and validated key enabling reagents for dozens of potential AD drug targets for which there were few reagents, but also developed new approaches to expedite target prioritization and organization, antibody characterization, and chemical probe discovery. The current target portfolio was assembled by evaluating prioritized understudied proteins by the AMP‐AD consortium and additional NIA‐supported AD consortia. Nominated targets were evaluated by calculating an unbiased AD risk score and then mapped to 19 biological domains (BDs) that describe and codify the different processes that are dysregulated in AD and prioritized based on multiple lines of evidence for overall AD‐risk. Targets that meet criteria for development are evaluated to identify a set of experimental reagents necessary for hypothesis testing.

**Result:**

We have prioritized more than 50 understudied targets for TEP development. For each understudied target, a TEP includes expression constructs, purified protein and methods, validated knockout cell lines, and antibody validation. For a subset of tractable targets additional TEP components include assay development, crystal structures, screening, and probe development. Advanced targets include SYK, DDX1, SDC4, ARHGEF2, and MDK.

**Conclusion:**

All data, protocols, reagent sets, and chemical tools will be made widely available on the AD Knowledge Portal with no intellectual property claims. For more information see www.treatad.org.

